# Exploring HTML5 Package Interactive Content in Supporting Learning Through Self-Paced Massive Open Online Courses on Healthy Aging: Mixed Methods Study

**DOI:** 10.2196/45468

**Published:** 2024-08-22

**Authors:** Pratiwi Rahadiani, Aria Kekalih, Diantha Soemantri, Desak Gede Budi Krisnamurti

**Affiliations:** 1 Center of e-Learning Cluster, Indonesia Medical Education and Research Institute Faculty of Medicine Universitas Indonesia Central Jakarta Indonesia; 2 Department of Community Medicine Faculty of Medicine Universitas Indonesia Central Jakarta Indonesia; 3 Department of Medical Education Faculty of Medicine Universitas Indonesia Central Jakarta Indonesia; 4 Department of Medical Pharmacy Faculty of Medicine Universitas Indonesia Central Jakarta Indonesia

**Keywords:** HTML5 package, H5P, students’ perspectives, students’ acceptance, massive open online courses, MOOCs, healthy aging, self-paced MOOC, student, perception, acceptance, opinion, attitude, MOOC, self-paced, self-guided, online course, online learning, geriatric, gerontology, gerontological, learning

## Abstract

**Background:**

The rapidly aging population and the growth of geriatric medicine in the field of internal medicine are not supported by sufficient gerontological training in many health care disciplines. There is rising awareness about the education and training needed to adequately prepare health care professionals to address the needs of the older adult population. Massive open online courses (MOOCs) might be the best alternative method of learning delivery in this context. However, the diversity of MOOC participants poses a challenge for MOOC providers to innovate in developing learning content that suits the needs and characters of participants.

**Objective:**

The primary outcome of this study was to explore students’ perceptions and acceptance of HTML5 package (H5P) interactive content in self-paced MOOCs and its association with students’ characteristics and experience in using MOOCs.

**Methods:**

This study used a cross-sectional design, combining qualitative and quantitative approaches. Participants, predominantly general practitioners from various regions of Indonesia with diverse educational backgrounds and age groups, completed pretests, engaged with H5P interactive content, and participated in forum discussions and posttests. Data were retrieved from the online questionnaire attached to a selected MOOC course. Students’ perceptions and acceptance of H5P interactive content were rated on a 6-point Likert scale from 1 (strongly disagree) to 6 (strongly agree). Data were analyzed using SPSS (IBM Corp) to examine demographics, computer literacy, acceptance, and perceptions of H5P interactive content. Quantitative analysis explored correlations, while qualitative analysis identified recurring themes from open-ended survey responses to determine students’ perceptions.

**Results:**

In total, 184 MOOC participants agreed to participate in the study. Students demonstrated positive perceptions and a high level of acceptance of integrating H5P interactive content within the self-paced MOOC. Analysis of mean (SD) value across all responses consistently revealed favorable scores (greater than 5), ranging from 5.18 (SD 0.861) to 5.45 (SD 0.659) and 5.28 (SD 0.728) to 5.52 (SD 0.627), respectively. This finding underscores widespread satisfaction and robust acceptance of H5P interactive content. Students found the H5P interactive content more satisfying and fun, easier to understand, more effective, and more helpful in improving learning outcomes than material in the form of common documents and learning videos. There is a significant correlation between computer literacy, students’ acceptance, and students’ perceptions.

**Conclusions:**

Students from various backgrounds showed a high level of acceptance and positive perceptions of leveraging H5P interactive content in the self-paced MOOC. The findings suggest potential new uses of H5P interactive content in MOOCs, such as interactive videos with pop-up questions, to substitute for synchronous learning. The study underscores the significance of tailored educational strategies in supporting the professional development of health care professionals.

## Introduction

Indonesia is one of the most populous countries in the world, with a population of about 268 million people. In 2021, there were 10.8% or around 29.3 million older adults in the population. By 2045, the ratio of older adults is expected to increase to 19.9% [1]. The rising number of older adults poses potential challenges and vulnerability in medical, psychological, economic, and social domains within this population. The aging process is unavoidable and can potentially bring various challenges in terms of health and quality of life [2]. Furthermore, it contributes to increasing mortality from noncommunicable diseases among older adults [3].

In response to this problem, the Indonesian government launched the 2016-2019 National Action Plan for Elderly Health to improve the quality of life of older adults through health service programs. However, this program has not been run optimally. The implementation of geriatric services in hospitals is still not realized. The policy exists and the implementation of the program is quite good, but the instrument for measuring it has not been understood and used by service providers [4]. According to the Central Bureau of Statistics data, Indonesia had 173,779 doctors and 2,287,142 health workers spread throughout the country in 2021 [5,6]. However, data from the Health Human Resources Information System in 2018 reported that only 23% of health community service centers had qualified health care professionals and 15% had none [7]. This indicates that the coverage of health services for older adults has not met expectations and is unevenly distributed [8]. Doctors and health care professionals need to be equipped with the knowledge and skills to ensure a healthy and independent older adult population.

Moreover, while geriatric medicine is a rapidly growing field within internal medicine, gerontological training is still limited across many health care disciplines today [9,10]. This highlights the importance of raising awareness about education and training in geriatric health care, ensuring health professionals are adequately prepared to address the needs of the older adult population. Therefore, education for health professionals and continuing education for practitioners is required to reframe medical care to meet the needs and personal quality of life goals of older adults [11].

Many health care professionals experience difficulties traveling to attend face-to-face continuing training due to their busy work schedules and their location in remote rural areas. However, time and travel restrictions during the COVID-19 pandemic catalyzed a shift from face-to-face to online education [12]. As a form of e-learning, massive open online courses (MOOCs) have emerged as an invaluable tool to address some of the training challenges experienced in developing countries by supporting content delivery that is efficient, cost-effective, and accessible [13]. MOOCs can provide training for health care professionals and increase the dissemination of information about public health issues to the public [14]. Skinner et al [15] found that health care professionals need MOOC content that is easy to adapt and share. Thus, MOOCs can effectively deliver learning materials in health care education and continuing education for practitioners.

Despite the many benefits of MOOCs, such as their open nature and enabling teachers to reach a large and diverse group of participants, there is a perception of social isolation regarding the lack of interaction between teachers and students and between students [16].

Furthermore, the diversity of participants makes it difficult to engage students, which further adds to the complexity of the students’ interactions with the course content [17], even though such interaction is the most common interaction form in MOOCs [18]. This challenges MOOC providers to develop innovative learning content that suits the needs and characters of users to increase student engagement and mastery of learning. The existing MOOC platforms use videos as the main information delivery method. The videos are presented in a 1-way format where students are passive viewers, making learning feel monotonous [18,19].

The use of the HTML5 package (H5P) to develop interactive learning content can make the class more interactive and fun and encourage self-directed learning. Moreover, H5P allows students’ learning outcomes to be recorded so that they can be used as evaluation material for instructors [20]. H5P is a simple and easy-to-use open-source technology without the need for plugins or Shareable Content Object Reference Model (SCORM) standards. The interactive learning content can be developed in formats such as interactive videos, course presentations, image hotspots, and branching scenarios [20,21].

Previous studies have demonstrated how H5P interactive content can support online learning environments, including blended learning, flipped classrooms, active learning, and virtual simulations. However, few studies have focused specifically on the use of H5P within Moodle-based MOOC platforms, especially in health profession education and continuing education [22-24]. Moreover, limited research exists on Indonesian students’ perspectives regarding their participation in self-paced MOOCs with interactive content H5P, especially in the context of healthy aging. This study explored students’ perceptions and levels of acceptance of H5P interactive content in a self-paced MOOC.

## Methods

### Design and Settings

The study used a combination of qualitative and quantitative approaches. The participants enrolled in a course comprised of 8 topics on the MOOC platform (Table 1). The course included a pretest, learning material, forum discussions, and a posttest on every topic. At the end of the course, we asked participants to complete a self-reflection task regarding their experience during the course. The course was developed using a self-paced learning method so participants could progress at their own pace. Learning material provided in the H5P took the form of interactive books and interactive videos. There were between 3 and 8 videos per topic in the module, with an average duration of around 12 minutes for each video, and each interactive video included pop-up questions. A survey was attached at the end of the course for students to answer questions on their acceptance of H5P interactive content and their views on whether it supports self-paced learning.

**Table 1:** Tittle and estimated duration to complete for each topic.

Topic	Tittle	Duration (hours)
1	The Role of Healthy Aging and Risk Factors That Can Lead to Non-Communicable Disease	2
2	Elderly and its Problems	2
3	Metabolic and Hormonal Aspects of Aging	2
4	Risk Factors and Cardiovascular Disease in the Elderly	4
5	Neurodegenerative Diseases in the Elderly and Prevention	2
6	The Role of Physical Activity as Prevention of Non-Communicable Diseases in the Elderly	3
7	The Role of Nutrition as Prevention of Non-Communicable Diseases in the Elderly	8
8	The Role of Social Support and Medical Funding in Overcoming Diseases in the Elderly	4

### Participants

Due to their scattered locations throughout Indonesia, the participants were recruited via email and WhatsApp (Meta Platforms). We identified potential participants through various educational networks and organizations dedicated to supporting students across Indonesia. Specifically, we leveraged our existing contacts within these networks and used databases of educational institutions. Additionally, participants were identified based on recommendations from local contacts familiar with the target demographics. Furthermore, to ensure the relevance of participants, we specifically targeted general practitioners practicing in diverse locations throughout Indonesia. The participants were enrolled in an online course on the MOOC platform from August to September 2021. There were 796 participants in the course. This study used purposive sampling as its sampling method. The sample was selected based on participants’ completion of the course. A total of 184 participants completed the course and were selected to participate and analyze in this study.

### Ethical Considerations

The ethics committee of the Faculty of Medicine, Universitas Indonesia-Cipto Mangunkusumo Hospital, has approved this study (KET-511/UNI2.F1/ETIK/PPM.00.02/2021). Informed consent was obtained from all participants involved in the study. Confidentiality and anonymity were preserved during data collection and processing.

### Data Collection

We retrieved data from the online questionnaire attached to the MOOC course. The questionnaires were adapted and modified from a variety of existing instruments [25-27]. The questionnaire was written in Bahasa Indonesia and included items on the demographic characteristics of students (age range, sex, education, and experience of MOOCs), their computer literacy rated on a 5-point Likert scale from 1 (bad) to 5 (excellent), students’ perceptions and acceptance of H5P interactive content rated on a 6-point Likert scale from 1 (strongly disagree) to 6 (strongly agree). The student perception questionnaire was adapted and modified from the study by Muthuprasad et al [27]. The open-ended question was used to emphasize findings from the students’ perception questionnaire. The researcher with experts was involved in reviewing the questionnaire items and validated it using content validation methods. Meanwhile, students’ acceptance was measured using the Technology Acceptance Model (TAM) adopted and modified from the study by Masrom [26], which consists of 2 variables—perceived ease of use and perceived usefulness. According to Davis [28], these 2 variables of TAM are the 2 main factors that influence behavioral intentions toward new technologies and they affect the actual use of the technologies [28]. The validity and reliability of the instrument were tested in the previous study. The result indicates that the validity test of students’ perceived ease of use variables ranged from 0.783 to 1.000, and perceived usefulness ranged from 0.731 to 0.977. Cronbach α values of perceived ease of use and perceived usefulness were 0.975 and 0.973, respectively. Scores greater than 0.7 indicate reliability and good internal consistency [29].

### Quantitative Analysis

We analyzed the data using SPSS (version 25.0; IBM Corp). Descriptive analysis was used to analyze students’ characteristics, perceptions, and acceptance of the H5P content. A bivariate correlation (Pearson correlation) was conducted to determine the relationships among predictor factors such as students’ computer literacy and their perceptions and acceptance of H5P interactive content in terms of perceived ease of use and perceived usefulness based on the TAM. Conditions for multicollinearity and homoscedasticity were met. No multicollinearity between independent and dependent variables was found in the preliminary study. The variance inflation factor values were all below 5. We investigated perceived ease of use, perceived usefulness, and students’ perception as dependent variables, and age, sex, education, computer literacy, and MOOC experience as independent variables.

### Qualitative Analysis

In the qualitative analysis, we identified themes related to students’ perceptions and opinions from the open-ended survey questions using a constant comparison process [30] and inductive analysis. First, the researchers read each response to gain a grasp of the information. Second, they broke the data into smaller units, coded and labeled the units according to the content they contained. Finally, categories and overarching themes were defined. During the analysis, the data were constantly compared back and forth between the current data and previous data that had been coded. All data analysis was completed manually using Microsoft Excel.

## Results

### Student Characteristics

**Figure 1:**
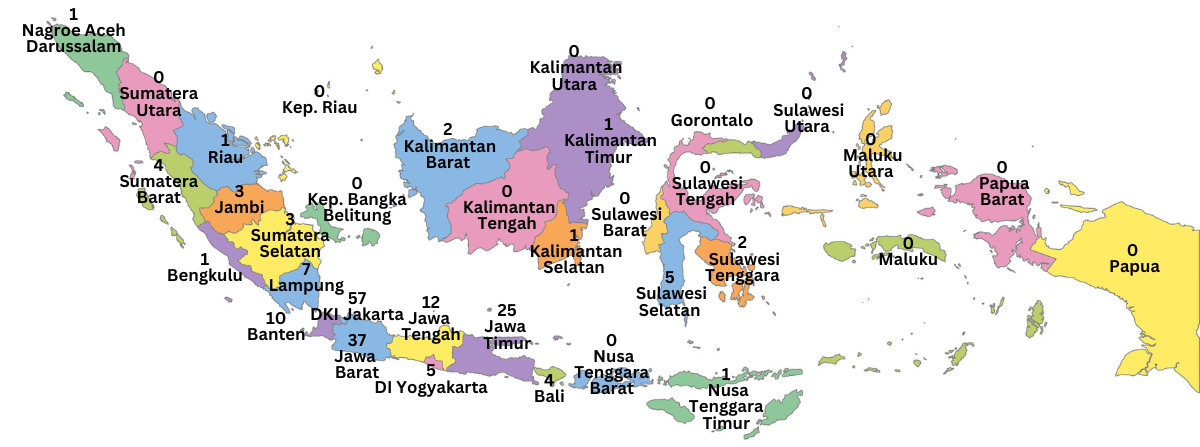
Characteristics of students based on the domicile area (adapted from Vemaps [31], which is published under Creative Commons Attribution (CC-BY) [32].

Table 2 presents all the detailed characteristics of students. The participants ranged in age from 36 to 45 years old, with 70.7% (n=130) female participants. Most participants live on Java Island and the rest are scattered throughout Indonesia (Figure 1 [31]).

The educational backgrounds of students were dominated by participants who held bachelor’s (40.2%, n=74) and master’s (42.9%, n=79) degrees. Most of the participants were doctors (66.8%, n=123). In terms of MOOC experience, most participants had no previous experience of taking MOOCs.

**Table 2:** Students’ characteristics (N=184).

Characteristics		Values, n (%)
**Age range (years)**		
	<25	33 (17.9)
	26-35	49 (26.6)
	36-45	63 (34.2)
	46-55	26 (14.1)
	>56	13 (7.1)
**Sex**		
	Male	54 (29.3)
	Female	130 (70.7)
**Domicile**		
	Nanggroe Aceh Darussalam	1 (0.5)
	Sumatera Barat	4 (2.2)
	Riau	1 (0.5)
	Kepulauan Riau	1 (0.5)
	Jambi	3 (1.6)
	Bengkulu	1 (0.5)
	Sumatera Selatan	3 (1.6)
	Lampung	7 (3.8)
	Banten	10 (5.4)
	Daerah Khusus Ibukota Jakarta	57 (31.0)
	Jawa Barat	37 (20.1)
	Jawa Tengah	12 (6.5)
	Jawa Timur	25 (13.6)
	Daerah Istimewa Yogyakarta	5 (2.7)
	Bali	4 (2.2)
	Nusa Tenggara Timur	1 (0.5)
	Kalimantan Barat	2 (1.1)
	Kalimantan Selatan	1 (0.5)
	Kalimantan Timur	1 (0.5)
	Sulawesi Selatan	5 (2.7)
	Sulawesi Tenggara	2 (1.1)
	Overseas	1 (0.5)
**Education**		
	High school	5 (2.7)
	Bachelor’s degree	74 (40.2)
	Master’s degree	79 (42.9)
	Doctoral degree	26 (14.1)
**Occupation**		
	Student	11 (6.0)
	Doctor	123 (66.8)
	Health worker	2 (1.1)
	Lecturer	48 (26.1)
**MOOC^a^ experience**		
	Yes	63 (34.2)
	No	121 (65.8)

^a^MOOC: massive open online courses.

### Computer Literacy (Self-Efficacy)

Computer literacy or computer self-efficacy of students who participated in the course are presented in Table 3. The mean value of computer literacy responses ranged from 3.92 to 4.28, and the SD ranged from 0.725 to 0.871. Overall, students’ computer literacy was good. The item on computer maintenance ability (installing programs, applications, or software) had the lowest mean value of 3.92 (SD 0.871), and the item related to browsing skills had the highest mean value of 4.28 (SD 0.751).

**Table 3:** Computer literacy of participants (N=184).

Statements	Values, mean (SD)
CL1—Ability to use computer	4.21 (0.725)
CL2—Basic computer operation skills (typing accuracy and speed, moving cursor)	4.24 (0.773)
CL3—Running computer programs, software, or applications	4.12 (0.759)
CL4—Computer maintenance ability (installing programs or applications)	3.92 (0.871)
CL5—Browsing skills	4.28 (0.751)

### Perception and Opinion of the Participant Toward H5P Interactive Content Usage

Table 4 shows the results of a descriptive analysis of students’ perceptions of H5P interactive content. The mean value of all responses falls between 5.18 and 5.45, and the SDs range from 0.605 to 0.861, indicating that students generally have a positive perception toward H5P interactive content leveraging. The results indicate that students perceived H5P interactive content as fun, easier, and more helpful in promoting understanding and improving learning outcomes, and more satisfying than material in the form of common documents and learning videos. Thus, students’ perceptions of how interactive content can replace synchronous learning had the lowest mean value of 5.18.

**Table 4:** Students’ perception toward H5Pa interactive content (N=184).

Statement	Values, mean (SD)	Range
SP1—I prefer the presentation of material in interactive form (interactive video and interactive book) than in the form of common documents or learning videos	5.45 (0.659)	3-6
SP2—Presentation of material in interactive form (interactive video and interactive book) provides a more enjoyable learning experience than presentation in the form of common documents or learning videos	5.43 (0.633)	3-6
SP3—Presentation of material in interactive form (interactive video and interactive book) makes it easier for me to understand learning compared to presentation in the form of common documents or learning videos	5.43 (0.605)	3-6
SP4—Presentation of material in interactive form (interactive video and interactive book) is more effective than presentation in the form of common documents or learning videos	5.38 (0.675)	1-6
SP5—Presentation of material in interactive form (interactive video and interactive book) is more effective than compared to presentation in the form of common documents or learning videos	5.36 (0.687)	1-6
SP6—Presentation of material in interactive form (interactive videos and interactive books) makes me more focused than presenting in the form of common documents or learning videos	5.38 (0.691)	1-6
SP7—I am more satisfied with the presentation of material in interactive form (interactive videos and interactive books) than in the form of common documents or learning videos	5.34 (0.786)	1-6
SP8—Presentation of material in interactive form (interactive video and interactive book) can replace synchronous learning	5.18 (0.861)	1-6

^a^H5P: HTML5 package.

The students’ perceptions and opinions about H5P interactive content were also explored in the open-ended questions supporting the findings from the perception assessment. According to the responses, students believe that H5P interactive content could increase focus and memory retention, as illustrated by the following quotes.

Overall, the learning content is very good. If possible, learning videos with pop-up questions are applied to all materials because they can increase focus and memory retention on related materials.

Female, 27 years old

…Giving interactive book material in approximately 15 minutes can maintain concentration. Moreover, having a quiz in the middle of a video can help me to stay focused.

Female, 45 years old

…Videos with pop-up questions really help to increase my attention when taking lessons…

Female, 28 years old

Furthermore, students also noted the advantages of interactive videos with pop-up questions. They reported that they provide a different and better learning experience than synchronous learning through Zoom (Zoom Video Communications), are good and interactive, and can cross-check their understanding during the learning process. So, students suggest adding more questions during the video, as highlighted in the quotes.

Increasing number of pop-up questions in the middle of learning materials…

Female, 30 years old

I suggest applying pop-up questions in the midst of all interactive videos, so that it will provide a different and better learning experience than learning through Zoom.

Male, 24 years old

I think interactive videos with pop-up questions are good and interactive. If possible, every video should have an interaction (pop-up questions) so we can cross-check our understanding during the learning process.

Male, 31 years old

### Students’ Acceptance of H5P Interactive Content

The findings presented in Table 5 indicate that students had a high level of acceptance of H5P interactive content. All 5 items measuring perceived ease of use and the 5 items measuring perceived usefulness recorded mean values above 5 points. This indicates that respondents found the H5P interactive content easy to use and effective for helping them learn on the MOOC.

**Table 5:** Students’ acceptance to H5P^a^ interactive content (N=184).

Statements			Values, mean (SD)		Range
**Perceived ease of use**					
	PEU1—I can easily access the interactive learning content available in this module	5.36 (0.663)		3-6	
	PEU2—The navigation provided in the interactive learning content in this module can be easily understood	5.28 (0.728)		3-6	
	PEU3—I can easily understand the instructions given on the interactive learning content in this module	5.38 (0.683)		3-6	
	PEU4—I can easily operate the interactive learning content in this module	5.37 (0.742)		2-6	
	PEU5—Overall interactive learning content is easy to use	5.45 (0.651)		3-6	
**Perceived usefulness**					
	PU1—The questions that arise during the learning video playback can increase my attention (attention) to the material presented	5.52 (0.627)		3-6	
	PU2—The interactive video presented was able to trigger my curiosity further about the material	5.41 (0.679)		3-6	
	PU3—The interactive video presented was able to make it easier for me to understand the material	5.40 (0.602)		4-6	
	PU4—The use of interactive learning content (H5P) allows me to better manage my study time	5.43 (0.658)		3-6	
	PU5—Overall, I find it helpful to have H5P interactive learning content	5.51 (0.572)		4-6	

^a^H5P: HTML5 package.

### H5P Interactive Content Acceptance and Student Performance in the MOOC

This study examined several factors, including age range, sex, education, MOOC experience, computer literacy, perceived ease of use, perceived usefulness, and students’ perception. The correlations between these factors are presented in Table 6. The results reveal a significantly negative correlation between computer literacy and both age and sex. In contrast, there is a significantly positive correlation between computer literacy and MOOC experience. Computer literacy had a positive impact on perceived ease of use, perceived usefulness, and students’ perception.

**Table 6:** Correlation among variables.

	1	2	3	4	5	6	7
Age range (years)	1	—^a^	—	—	—	—	—
Sex	0.237^b^	1	—	—	—	—	—
Education	0.587^b^	0.182^c^	1	—	—	—	—
MOOC^d^ experience	–0.226^b^	–0.315^b^	–0.034	1	—	—	—
Computer literacy	–0.441^b^	–0.172^c^	–0.135	0.277^b^	1	—	—
Perceived ease of use	–0.124	–0.050	–0.112	0.065	0.395^b^	1	—
Perceived usefulness	–0.079	0.054	–0.154^c^	0.024	0.275^b^	0.795^b^	1
Students’ perception	–0.140	0.007	–0.119	0.037	0.339^b^	0.662^b^	0.752^b^

^a^Not applicable.

^b^Correlation is significant at 0.01 level (2-tailed).

^c^Correlation is significant at 0.05 level (2-tailed).

^d^MOOC: massive open online courses.

## Discussion

This study explored students’ perceptions and acceptance of H5P interactive content. We found that students have positive perceptions and acceptance, particularly of interactive books and videos with pop-up questions. The lack of instructor presence and student-instructor interactions in a self-paced MOOC potentially hampers students’ commitment and intention to commit to further learning. Therefore, student-content interaction needs to be maximized [33]. Student-content interaction may include reading information, watching videos, completing assignments, interacting with computer-based multimedia, using simulations, searching for information, and working on projects [34]. According to Kuo et al [35], students reported that interaction with the course content increased their satisfaction with the course. MOOCs offer openly accessible online participation, meaning participants can be from diverse backgrounds. The data on the characteristics of participants in this study show various backgrounds in terms of age, sex, education, job and employment status, computer literacy, previous knowledge of the material or topic, and experience with MOOCs. This indicates that such learning content should be designed to meet the diverse needs of students.

Our findings indicate that students from diverse backgrounds showed a high level of acceptance of H5P interactive content in the self-paced MOOC. They preferred H5P interactive content over traditional teaching methods because it is more fun, easier, more effective, more helpful in facilitating learning and improving learning outcomes, and more satisfying. Moreover, students also found that H5P interactive content (interactive video) provided a different and better learning experience than synchronous learning. Previous research suggests that more satisfying experiences will lead to better learning outcomes [36].

Another interesting finding emerged from the open-ended question analysis. We found that students perceived that pop-up questions during a video increased their focus and memory retention. They even suggested incorporating more questions during the video. This feedback has led to another research question regarding the optimum number of questions needed to make learning more effective and less monotonous. While previous studies have explored the relationship between the length of the intervals between questions and the rates of correct answers [37], the ideal number of questions has yet to be investigated.

Al-Adwan [38] defined computer self-efficacy as individuals’ beliefs about their computer abilities and skills. Research indicates that computer skills are one of the critical factors in successful e-learning. Moreover, many empirical studies have demonstrated that computer self-efficacy is a key predictor of perceived ease of use and usefulness in e-learning [34,35]. This aligns with the findings of this study, which indicated computer self-efficacy had a significantly positive impact on participants’ perception and acceptance of the perceived ease of use and usefulness of H5P interactive content in the self-paced MOOC. Students with high levels of computer self-efficacy are less likely to be discouraged by difficulties [39]. In contrast, students with low computer self-efficacy are more likely to give up when faced with challenges. To improve the learning experience, we suggest providing a step-by-step written or video tutorial to familiarize people with lower computer literacy. Students’ prior experience of MOOCs positively correlated with computer literacy but had no significant impact on their perception of ease of use and usefulness of H5P interactive content. This contrasts with the findings of Wang et al [40], which demonstrated that students’ prior experiences were positively associated with their satisfaction with their current learning experience.

This study has some limitations. First, the sample size is small and the participants share individual opinions. We used open-ended questions at the end of the questionnaire to provide more detailed responses to the survey responses; however, we did not follow up with the participants to confirm their answers. This survey is subject to individual variations among participants; their personal circumstances, work, and educational routines might have influenced their answers. Moreover, this study is also limited to the type of H5P interactive content used, namely H5P interactive video. Nevertheless, these study findings can form the basis for a pilot project to further analyze the potential of H5P interactive content to improve the interaction and engagement of students in self-paced MOOCs. We found that H5P interactive content videos with pop-up questions can substitute for synchronous learning; however, further study is necessary to examine its impact on learning outcomes.

In conclusion, this research suggests the use of H5P interactive content, especially interactive books with pop-up questions, can potentially substitute for synchronous learning in the context of self-paced MOOCs. Positive perceptions and high-level acceptance by students toward the use of H5P interactive content suggest that it is suitable for diverse participants of MOOCs from various regions of Indonesia, with diverse educational backgrounds and age groups. Future research should compare students’ learning performance in self-paced MOOCs with and without H5P interactive content.

## Abbreviations

H5P: HTML5 Package
MOOC: Massive Open Online Course
SCORM: Shareable Content Object Reference Model
TAM: Technology Acceptance Model

## Data Availability

All data generated or analyzed during this study are included in this published paper.
